# Earlier flowering did not alter pollen limitation in an early flowering shrub under short-term experimental warming

**DOI:** 10.1038/s41598-017-03037-9

**Published:** 2017-06-05

**Authors:** Cheng-Chen Pan, Qi Feng, Ha-Lin Zhao, Lin-De Liu, Yu-Lin Li, Yu-Qiang Li, Tong-Hui Zhang, Xiao-Ya Yu

**Affiliations:** 1 Northwest Institute of Eco-Environment and Resources, Chinese Academy of Sciences, Lanzhou, 730000 China; 2 Key Laboratory of Ecohydrology of Inland River Basin, Chinese Academy of Sciences, Lanzhou, 730000 China; 3 College of Life Sciences, Ludong University, Yantai, 264025 China; 4 School of Tourism and Resource Environment, Qiannan Normal University for Nationalities, Duyun, 558000 China

## Abstract

In animal pollinated plants, phenological shifts caused by climate change may have important ecological consequences. However, no empirical evidence exists at present on the consequences that flowering phenology shifts have on the strength of pollen limitation under experimental warming. Here, we investigated the effects of experimental warming on flowering phenology, flower density, reproductive success, and pollen limitation intensity in *Caragana microphylla* and evaluated whether earlier flowering phenology affected plant reproduction and the level of pollen limitation using warmed and unwarmed open top chambers in the Horqin Sandy Land of Inner Mongolia, northern China. The results of this study indicated that artificial warming markedly advanced flower phenology rather than extending the duration of the flowering. Additionally, warming was found to significantly reduce flower density which led to seed production reduction, since there were insignificant effects observed on fruit set and seed number per fruit. Experimental floral manipulations showed that warming did not affect pollen limitation. These results revealed the negative effects of advanced phenology induced by warming on flower density and reproductive output, as well as the neutral effects on reproductive success and pollen limitation intensity of long surviving plants.

## Introduction

Global mean temperature has risen by 0.85 °C since the 1880s, and is predicted to increase an additional 1.8–4 °C by the end of this century^1^. The effects of global warming on ecosystem structure and function have received increasing attention world-wide. Global warming has resulted in a change in flowering phenology and/or a decline in flower production^2^, leading to a change in reproductive output of plants. It has been frequently reported that the time of flowering could be either advanced or delayed by artificial warming^2–6^. These phenological shifts could potentially increase pollen limitation^7, 8^, which results in reduced fruit and seed sets caused by a scarcity of pollen receipt^9^. Many previous studies have recorded a shift in the reproductive output in response to warming^2, 10, 11^. However, the effect of the shift in flowering phenology induced by experimental warming on pollen limitation intensity has not been empirically examined. Shifts in flowering time may expose species to novel abiotic and biotic environments, and these components may interact to determine plant reproduction and pollen limitation^10^. It has been previously documented that plant populations suffering from pollen limitation experience disruptions in seed production^12^. Pollen limitation induced reproductive failure may lead to a reduction in population growth rates and their long-term viability^13, 14^. Severe and consistent pollen limitation may even cause local extinctions^15^. It has been predicted that climate warming may drive thousands of plant species to the brink of extinction over the next century, due to changes in the timing of their life cycles, such as flowering phenology^16, 17^. Therefore, research concerning the reproductive success and pollen limitation of endemic plants living in a warmer world is essential to the understanding and prediction of the consequences of the climate warming-mediated timing of plant life cycles, as well as the changes in the conservation of plant populations.

In this study, we report the effects of a short-term warming experiment in the Horqin Sandy Land of Inner Mongolia, northern China. This area is one of the regions that has been predicted to experience “a much more rapid than the average” increase in surface temperature in the future^18^. In order to test whether climate warming has a significant effect on flower phenology and increases pollen limitation, we carried out an artificial warming experiment using open top chambers (OTCs). In the second year after warming, we performed a pollen supplementation experiment, and examined flowering onset, end, duration, as well as fruit set and seed number per fruit in *Caragana microphylla*. *C. microphylla* is a long-lived deciduous shrub in the Horqin Sandy Land, northern China. It is the first and the only floral resource available to flower visitors for several weeks in the Horqin Sandy Land, northern China. Therefore, both flowering onset and duration are important factors determining the food supply for flower visitors. This is particularly true for the known pollinators, such as *Xanthosaurus remota* and *Megachile desertorum*, as well as nectar thieves, such as *Amegilla parhypate* and *Liothyrapis* sp. (C. Pan, pers obs), bees that emerge early in the season. *C. microphylla* is self-compatible, but pollinators are essential for successful pollination^19^; therefore, *C. microphylla* may be particularly vulnerable to climate change.

The main goal of this study was to test the hypothesis that the temperature-driven flower phenology in an early flowering species of *Caragana* had a significant effect on the intensity of pollen limitation. In this sense, we proposed that: (1) climate warming would shift the flowering phenology of *C. microphylla*, and (2) plants would experience greater pollen limitation due to warming and therefore poorer reproductive success. The measurements of the variations in reproductive output and pollen limitation intensity with flowering phenology will provide a basic understanding of the potential temporal responses to climate change.

## Results

Plant flowering phenology was substantially advanced by warming. The first and last flowering dates were 6.6 and 6.4 days earlier in the warmed OTCs, respectively, when compared with control chambers, which indicated that the simulated warming did not alter the duration of flowering (Table 1).

**Table 1:** Means (±SE) of flowering onset, end, duration (Julian day), and flower density (number of flowers per cm of stalk length) of *Caragana microphylla* in both warmed and unwarmed open-top chambers (OTCs).

	Unwarmed OTCs	Warmed OTCs	*p*
Flowering onset time	137.6 ± 2.3	129.2 ± 2.3	0.03
Flowering end time	151.8 ± 1.6	146.6 ± 0.4	0.01
Duration of flowering	17.3 ± 2.2	17.4 ± 2.6	0.96
Flower density	0.82 ± 0.01	0.60 ± 0.04	0.002

The significance level of the difference in the parameters between warmed and ambient OTCs is denoted by *p* values.

**Figure 1:**
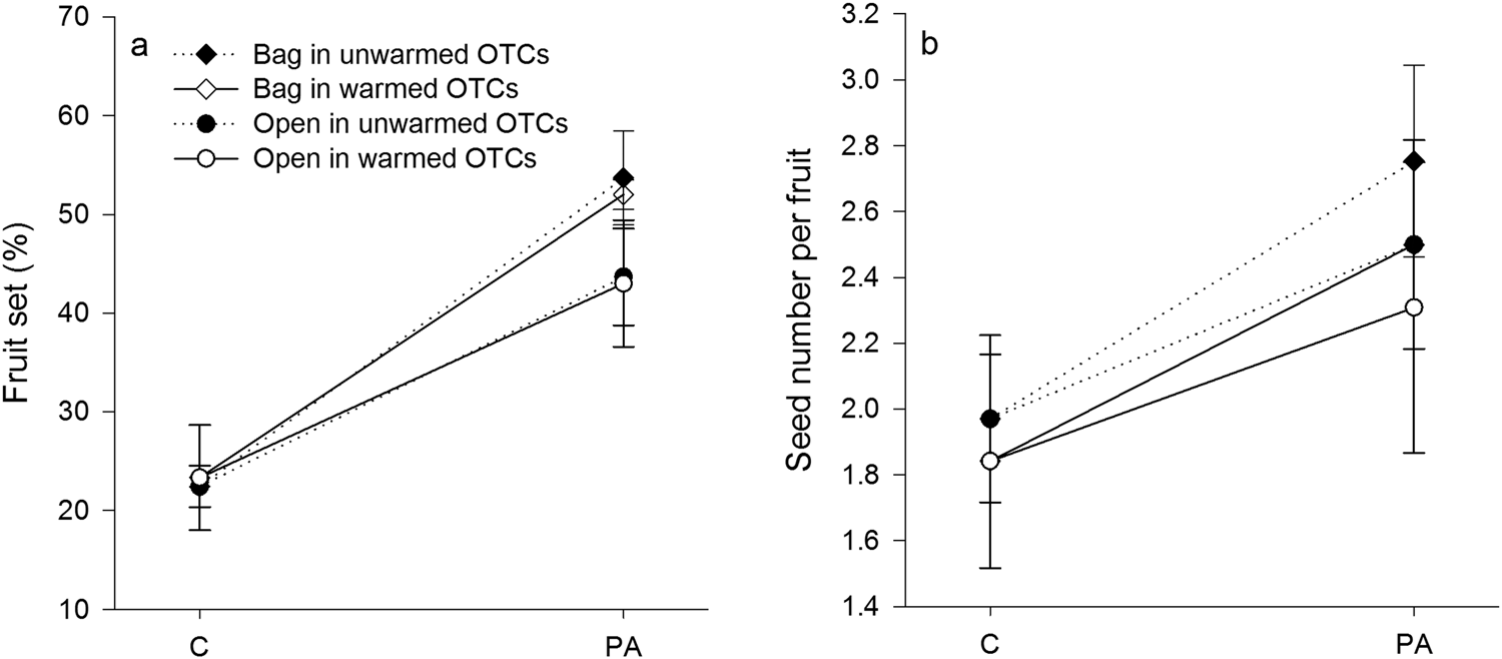
The effects of pollination treatments (control [C] vs. supplemental hand pollination [PA]) and warming (unwarmed vs. warmed) on (**a**) fruit set, and (**b**) seed number per fruit in *Caragana microphylla*.

Warming significantly reduced flower density (Table 1), but no effects were observed on fruit set and seed number per fruit (Fig. 1). These findings indicated a decline in seed production per unit area. Across all the warmed and unwarmed OTCs combined, pollen supplementation did not affect seed number per fruit (Fig. 1b). No significant difference was found in fruit set between the supplemental pollen treatments (Bag and Open; warmed OTCs: *F* = 0.64, *p* = 0.45; unwarmed OTCs: *F* = 2.11, *p* = 0.19; Fig. 1a). Significantly greater fruit set in the supplemental pollen treatments (Bag and Open) were detected compared to the Control (Fig. 1a), indicating pollen limitation. However, pollen limitation indices were not affected by the warming treatment (Open: *F* = 0.004, *p* = 0.95; Bag: *F* = 0.05, *p* = 0.96). In the warmed chambers, the mean pollen limitation indices in the Open and Bag treatments were 45.9% and 56.2%, whereas those in the ambient chambers were 45.1% and 55.7%, respectively.

## Discussion

Phenological shifts in plants under climate warming could potentially increase pollen limitation^7, 8^. Here, we provide experimental evidence inconsistent with this hypothesis. Our results showed that warming significantly advanced flowering phenology, and reduced flower density. On the other hand, fruit set, seed number per fruit, and pollen limitation were largely unaffected.

As an early flowering species in spring, the advanced onset of flowering was consistent with some species^2, 4, 6^, but differed from some other species that demonstrated delayed onset of flowering under warmed conditions^4, 6, 10^. These results support our initial hypothesis that short-term warming advances flowering phenology. This advance may simply be because our experiment advanced the date of spring soil thawing after the warmed OTCs were set up. The accumulated temperature for blooming reached earlier in the warmed chambers. Our results showed that the first and last flowering dates were very sensitive to the simulated warming, while the flowering duration was not. As shown above, simulated warming merely altered flowering, rather than prolonging it. This differed from the studies that were conducted in subalpine meadows, where advanced flowering led to extended flowering duration^20^. Therefore, flowering time responses to warming will vary with climatic regions^4^. A simulated warming experiment conducted in North America was found to significantly shorten the duration of flowering in some species, but prolonged it in others^4^. These findings indicated that experimental warming induced shift in flowering duration was species-specific^21^. Further work is needed to illustrate potential mechanisms for flowering duration variation under climate warming.

Flowering phenology is an important determinant of plant reproductive success^22^. However, in our case, fruit set and seed number per fruit were generally unaffected by changes in flowering phenology. These results were consistent with some terrestrial species^2, 11^. Fruit set and seed number per fruit under climate warming are also dependent on post-pollination events leading up to fertilization^23^. In regards to fruit set and seed number per fruit for *C. microphyll*a found under experimental warming, a future warmer world would unlikely have negative effects on pollination and fertilization success for this desert species. On the other hand, many studies showed that geitonogamy may significantly reduce the cross-pollination success^24, 25^. However, this may not occur in *C. microphylla* since no significant difference was found in reproductive success between Open and Bag in both types of OTCs. As previously detailed, the difference in reproductive output can only be attributed to the difference in the number of flowers. In our study, flower density appeared to be a more sensitive index, and diminished with the simulated warming of *C. microphylla* in the Horqin Sandy Land, northern China. Overall, these results demonstrated that experimental warming caused a decrease in the reproductive output of the studied shrub species, indicating that climate warming would negatively affect the reproduction and recruitment potential of *C. microphylla*, at least in the Horqin Sandy Land, China. The reduced seed production which resulted from the decreased flower production in the warmed OTCs may be due to the weakened or abated effects of vernalization under warming^2, 26^. Our findings obtained in a sand ecosystem contrasted with those obtained in alpine, arctic and high altitude ecosystems, where experimental warming has been observed to exert either positive^27–30^ or neutral^21, 31, 32^ effects on flower and seed production. Therefore, the current knowledge of climate change effects on plant biology should consider the different impacts across regional gradients of climate change^11^.

Our results showed that for the examined shrub, no obvious changes in pollen limitation index in fruit set and seed number per fruit were observed between warmed and unwarmed chambers. These observations suggested that the level of pollen limitation was largely unaffected by experimental warming for this species. These results were inconsistent with previous studies on reproductive output in Mediterranean shrubs^11^, and were inconsistent with the hypothesis that plants under experimental warming would suffer greater pollen limitation. Indeed, our results clearly showed that the decrease in reproductive output in the studied species (*C. microphylla*) was primarily due to a decrease in flower density, which cannot be interpreted in terms of pollen limitation.

In conclusion, the results demonstrated that the shift in flowering phenology induced by short-term experimental warming did not affect plant reproductive success and pollen limitation intensity in the early flowering shrub *C. microphylla*. The decline in seed production due to flower density decreasing may contribute to the decrease in dominance of the studied species in plant communities in the long run. The observed effects of warming in this study will benefit from further long-term, multi-year studies that can establish the effects of warming on the degree and extent of the inter-annual variabilities in plant flowering phenology and reproductive success.

## Materials and Methods

### Study species and field site

*C. microphylla* is a leguminous, spinose, and deciduous shrub that occurs as a main component in forb-steppe and semi-desert steppe^33^. It is the most dominant species of the 12 *Caragana* species found on the Mongolia steppes^34^. Plants can reach 3 m in height, and produce large yellow flowers. *C. microphylla* blooms massively during May in the study area. Additionally, it is androgynous, and belongs to the melittophilae cross pollinated plants.

Our study was conducted in the Naiman Desertification Research Station (Chinese Academy of Sciences), located in the southwestern end of the Horqin Sandy Land, Inner Mongolia, China (42°55′5″N, 120°41′49″E, 371 m asl). This region has a continental temperate climate with semi-arid and monsoon periods, characterized by a dry and windy winter and spring, a warm and comparatively wet summer, and a short, cool autumn. The mean annual temperature is 6.8 °C, with minimum and maximum monthly means of −13.2 °C and 23.5 °C in January and July, respectively. The annual mean precipitation is 366 mm (70% of which occurs during June to August). The mean wind speed is 4.3 m s^−1^, with occasional occurrences of gales ≥ 20 m s^−1^ in winter and spring, when the vegetation cover is lowest and the soil is driest^35^.

### Experimental design

Ten plants of similar size (160–180 cm in height) were deliberately selected (at a minimum spacing of 5 m) in a fenced, flat area of approximately 2500 m^2^ in late September 2015. Five plants were assigned to warmed OTCs (sides covered with transparent polycarbonate sheets) and 5 to unwarmed OTCs (sides covered with 425-μm-mesh white nylon screens). The OTCs were hexagonal, and measured 2 m in height, with a top diameter of 2.4 m, and a bottom diameter of 3.0 m with an individual center. These two types of OTCs were referred as the warmed chambers, and the unwarmed, ambient, control chambers, respectively. Bees were the main pollinators of *C. microphylla*. The unwarmed OTCs were installed as the same barrier for these bee pollinators as the warmed OTCs.

The measurements (Thermochron iButton DS1921G at centers of the OTCs) from October 2015 to September 2016 showed that soil temperature at a 5 cm depth and air temperature 20 cm above ground surface in the warmed OTCs were increased by 0.8–1.8 °C when compared with the unwarmed OTCs (Table 2).

**Table 2:** Means (±SE) of the temperature in warmed and unwarmed open-top chambers (OTCs).

		Soil surface (°C)	20 cm aboveground (°C)
Non-growing season (October 2015–March 2016)	In warmed OTCs	−0.5 ± 0.3	−2.6 ± 0.2
	In ambient OTCs	−2.3 ± 0.5	−3.6 ± 0.2
Growing season (April 2016–September 2016)	In warmed OTCs	23.6 ± 0.4	22.9 ± 0.1
	In ambient OTCs	22.8 ± 0.2	21.5 ± 0.2
Annual mean (October 2015–September 2016)	In warmed OTCs	12.6 ± 0.3	14.8 ± 0.1
	In ambient OTCs	11.3 ± 0.3	13.6 ± 0.1

### Flowering phenology

For each plant, we recorded the start and end of flowering to characterize flowering phenology. The flowering status of selected plants was recorded every second day. We defined flowering start as the day when the first flower opened, and flowering end as the day when the last flower wilted. We calculated flowering duration for individual plant as the number of days between an individual plant’s first and last flower.

### Pollen limitation

To investigate the intensity of pollen limitation and the consequences of geitonogamy for reproductive success, we applied three pollination treatments on each plant: (1) Control—flowers were unbagged and left to be naturally pollinated; (2) Bag treatment—flowers were bagged with nylon mesh pollination bags (20 × 45 cm, < 0.1 mm mesh light), and hand pollinated with outcross pollen; and (3) Open treatment—flowers were unbagged and allowed to be naturally pollinated. Meanwhile, flowers received hand pollinated outcross pollen as in the Bag treatment. Because plants can produce thousands of flowers, applying treatments to all the flowers on plants was impossible. As an alternative, we applied each treatment to all the flowers over 2–3 randomly selected stalks per plant. Then, the selected stalks’ lengths were measured. The flowers on each stalk were counted and the data were expressed as the number of flowers per cm of stalk length.

The study plots were visited every second day during the entire flowering period. All the open flowers on the selected branches for pollen added treatment were hand pollinated with outcross pollen on each visit. Since the flowers remained open for approximately five days, this protocol ensured that all the flowers received supplemental hand pollination at least once. Pollen was collected within a radius of 50 m from the focal plant. The flowers used for the pollen added treatment had newly opened, and were pollinated by hand from 9:00 am to 15:30 pm.

In late June, we counted the number of experimental flowers that had produced fruits to obtain fruit set (proportion of experimental flowers setting fruit) for each plant and treatment. In early July, we collected ripe fruits and dissected to count the number of seeds per fruit. A plant is considered resource limited if the mean fruit set or seed number per fruit cannot be elevated by hand pollination, whereas a plant is considered pollen limited if the mean fruit set or seed number per fruit are elevated by hand pollination^36^. Pollen limitation index (PL) was calculated as: PL = 1 − C/PA, where C and PA are the fruit set, or the seed number per fruit of the control and pollen added treatments (Bag and Open treatments), respectively^36^. PL values were calculated for each individual plant used in the experiment, and therefore, they were expressed at the plant level. The pollen limitation index ranges from 0 (indicating no pollen limitation) to 1 (indicating the highest pollen limitation).

### Data analysis

All data were averaged for each treatment on each individual in each OTC. The results were expressed as the mean ± standard error, SE. We used one way ANOVA to examine the effects of simulated warming on the starting and ending times of flowering, duration of flowering, flower density, fruit set, seed number per fruit, and pollen limitation intensity. The analyses were performed using DPS software. All *p*-values were considered significant at a significance level of 0.05.
